# Effects of soluble dietary fiber on glycolipid metabolism in gestational diabetes mellitus: study protocol for a randomized controlled clinical trial

**DOI:** 10.1186/s13063-025-09080-6

**Published:** 2025-09-24

**Authors:** Yiming Wang, Huacai Yuan, Ruyue Jiang, Keqing Jia, Xiaoping Ding, Ping Gu, Jianping Sun

**Affiliations:** 1 School of Public Health, Qingdao University, Qingdao, China; 2 Department of Clinical Nutrition, Qingdao Municipal Hospital, Qingdao, China

**Keywords:** Gestational diabetes mellitus, Dietary fiber, Inulin, Xylose oligosaccharides, Clinical trial

## Abstract

**Background:**

Accumulating evidence suggests that additional dietary fiber supplements may significantly improve glycolipid metabolism and pregnancy outcomes in individuals with gestational diabetes mellitus (GDM). However, the therapeutic effects of xylose oligosaccharides and inulin (XOS inulin) in pregnant women have not been investigated. Moreover, the underlying mechanism behind the therapeutic effects of this type of dietary fiber is not clear. Our study aims to assess the effects of daily XOS inulin supplementation on glycolipid metabolism and elucidate the therapeutic mechanism through gut microbiota analysis.

**Methods:**

This study is an 8-week, parallel-design, open-label, three-arm, single-center randomized controlled trial. Eligible participants were pregnant women between 24 and 28 weeks of gestation, and they were diagnosed with GDM through an oral glucose tolerance test (OGTT). The participants in the three groups will receive nutrition education alone, nutrition education plus XOS inulin (XOS 2 g and inulin 10 g) 12 g/day, or nutrition education plus XOS inulin 24 g/day. Measurements will be taken at baseline, week 4, and week 8. The primary outcome will be the change in glycosylated serum protein (GSP), and the key secondary outcomes include changes in fasting glucose, fasting insulin (FINS), 2-h postprandial plasma glucose (2 h-PPG), HbA1c, total cholesterol (TC), triglycerides (TG), LDL cholesterol (LDL-C), HDL cholesterol (HDL-C), and changes in the gut microbiota.

**Discussion:**

This study will evaluate the therapeutic effects of XOS inulin supplementation on glycemic control, lipid metabolism, gastrointestinal function, and perinatal outcomes in GDM patients and their offspring. It also provides insight into the potential role of the gut microbiome as a target for enhancing the therapeutic efficacy of emerging treatments for GDM. All participants will receive comprehensive GDM nutrition education, promoting sustainable dietary modifications that optimize maternal metabolic health and fetal outcomes.

**Trial registration:**

Chinese Clinical Trial registry ChiCTR2200060117. Registered on 19 May, 2022.

**Supplementary Information:**

The online version contains supplementary material available at 10.1186/s13063-025-09080-6.

## Background and rationale {6a}

Gestational diabetes mellitus (GDM) is a state of hyperglycemia, discovered during pregnancy via routine screening between 24 and 28 weeks of gestation [1]. The results of prospective studies indicate that GDM can pose a series of Health risks, such as preterm delivery, macrosomia, cesarean, and type 2 diabetes, to offspring or mothers [2, 3]. According to the meta-analysis of Ye et al., the total incidence of GDM in Mainland China is 14.8% [4]. The incidence of GDM in older mothers (> 35 years) in China is 26.7%, which is approximately twice as high as that reported in younger women [4]. Since the number of elderly pregnant women is increasing, effective therapeutic management is urgently needed to reduce the risk of long-term chronic diseases.

As guidelines recommend, 70–85% of women with GDM can control their blood glucose in the normal range by changing their lifestyle [5]. As an important part of lifestyle management, dietary nutrition therapy is needed to improve outcomes for women and their offspring. Considering the different dietary patterns between China and Western countries, limiting carbohydrate intake was recommended by the Chinese Medical Association (CMA) [6]. Another suggestion is to consume enough dietary fiber (up to 28 g/day), which is supported by American doctors and their Chinese colleagues [6]. Some studies support the idea that the intake of large amounts of dietary fiber can reduce the risk of GDM [7, 8]. However, only a few studies have explored the effects of additional soluble dietary fiber supplements in treating GDM, and the therapeutic effects of soluble fiber remain controversial [9].

Inulin and xylose oligosaccharides (XOS) are two types of soluble fibers. Only one study investigated the effects of pure inulin supplementation in treating GDM; the decrease in HbA1c in the experimental group was greater than that in the control group, but the difference in the decrease of fasting glucose level between the two groups was not significant [10]. Daily inulin intake may have therapeutic effect on GDM. In another small trial, consuming XOS 6 g/day significantly reduced fasting blood glucose of pregnant women with high risk factors for GDM, but the difference in GDM incidence between the two groups was not statistically significant. It is reported that XOS promotes the proliferation of bifidobacteria at a rate 20 times higher than other functional oligosaccharides. The mechanism underlying these positive effects involve changes in the gut microbiota caused by XOS [11]. As a novel food, XOS is considered safe under the proposed uses and use levels [12]. Previous study reported that no diarrhea episodes occurred on the first day of consumption of 3–5 g/day of XOS, but diarrhea occurrence on the first day of consumption of 10–12 g/day XOS was 18–20% [13]. A survey of Chinese women with GDM during their second trimester revealed that their daily dietary fiber intake was found to be only 11.7 ± 4.7 g, and there remains a significant gap between this and the Chinese Nutrition Society’s recommended daily dietary fiber intake of 30–35 g [14]. Based on the aforementioned evidence, we proposed that supplementing with a mixture of inulin and XOS to meet the recommend dietary fiber intake may have a beneficial therapeutic effect in pregnant women with GDM. However, no clinical studies to date have specially investigated this particular intervention.

Our study is to assess the effects of mixed inulin and XOS (XOS inulin) supplementation on glycolipid metabolism and the gut microbiome. The evidence generated from this trial will inform us whether daily XOS inulin supplementation is a viable new dietary approach to treat GDM.

### Explanation for the choice of comparators {6b}

To determine whether a dose–response relationship exists between the dose of XOS inulin and blood glucose metabolism, two supplement doses, 12 g/day and 24 g/day, will be used in the two experimental groups.

### Objectives {7}

We hypothesize that daily XOS-inulin supplementation improves glycolipid metabolism in GDM patients via modulation of gut microbiota. We designed this trial to test the efficacy of daily XOS inulin supplementation after the diagnosis of GDM for improving glycolipid control and changing the gut microbiome compared with standard dietary education. We also want to study the effects of XOS inulin supplementation on pregnancy outcomes.

### Trial design {8}

**Fig. 1:**
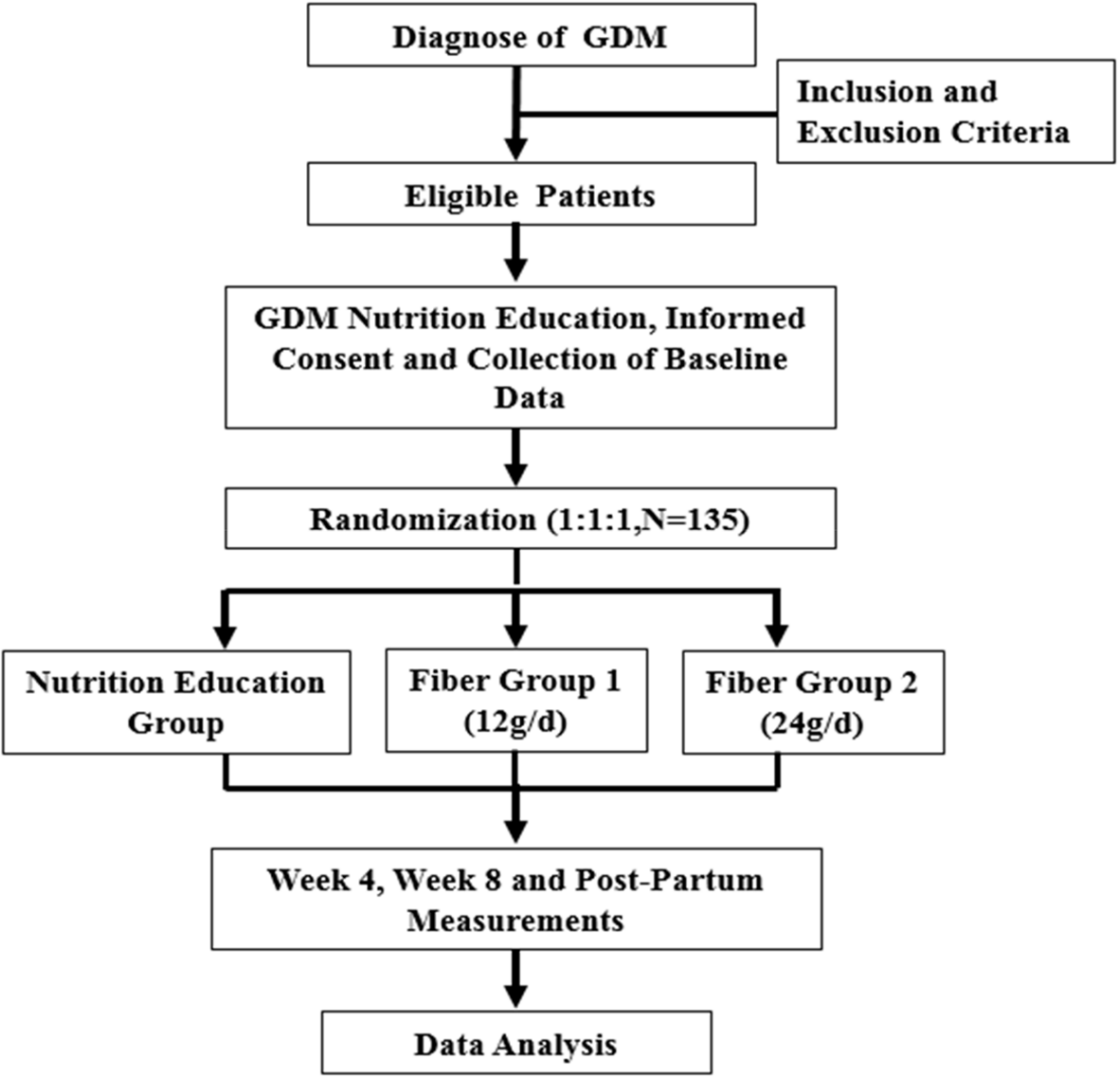
Flow chart of trial design, participant recruitment, and follow-up (GDM, gestational diabetes mellitus)

This study is an 8-week, parallel design, open-label, three-arm, single-center randomized controlled superiority trial. The participant flow through the trial is described in Fig. 1. The study protocol was designed and developed in accordance with the Standard Protocol Items: Recommendations for Interventional Trials (SPIRIT) 2013 checklist [15] (see Additional file 1). This trial was registered in the Chinese Clinical Trial registry: ChiCTR2200060117 (May 19, 2022).

### Study setting {9}

This trial will be carried out at the obstetric clinic of Qingdao Municipal Hospital, Qingdao, China.

### Eligibility criteria {10}

The inclusion criteria are as follows: (1) Pregnant women between 24 and 28 weeks of gestation, who were diagnosed with GDM through a routine 75 g of oral glucose tolerance test (OGTT) following the CMA diagnostic criteria if one or more values reached the following levels: fasting glucose ≥ 5.1 mmol/L, 1 h ≥ 10 mmol/L, and 2 h ≥ 8.5 mmol/L [13], (2) participants with singleton pregnancies, (3) patients treated with medical nutritional therapy, without insulin or other antidiabetic drugs, and (4) patients who signed an informed consent or whose guardian signed for them.

Participants will be excluded if they meet the following criteria: (1) were hospital inpatient with severe diseases; (2) had pregnancy induced diabetes mellitus; (3) had GDM and accepted hypoglycemic drug therapy or insulin injection; (4) did not receive prenatal care at Qingdao Municipal Hospital; (5) have consumed dietary fiber, probiotics, or prebiotic products in the past month; (6) have a history of surgery that would result in a change in the typical structure of the gastrointestinal tract, or have organic lesions of the intestine, or acute or chronic intestinal obstruction; and (7) have diseases that make them unsuitable for participation in this clinical trial.

### Interventions {11a}

All participants in the three groups will receive individualized dietary advice in line with updated guidelines issued by the ADA and CMA [5, 16]. First, women will be encouraged to eat at least 1600 cal daily to reduce the incidence of ketosis. Second, participants will be asked to consume a minimum carbohydrate intake of 175 g per day, mostly from low glycemic sources, distributed across the day over three meals and three snacks. Third, the intake of milk (500 g/day) and the amounts of fish, poultry, eggs, and lean meat should be increased by 50 g/day. We will also list the specific types of food that are suitable to eat. GDM participants will also be equipped with other effective skills, such as exercising, performing self-blood glucose monitoring, and keeping daily food diaries. All education will be conducted by experienced registered dietitians.

After collecting baseline data, women in one of the two fiber groups received two or four boxes of soluble dietary fiber (Nutrasumma, Qingdao Nutrasumma Health Technology Co., Ltd.) every month. Each box contains 15 bags (12 g/bag) of soluble dietary fiber (XOS inulin). The dietary fiber can be dissolved in warm water and has a mildly sweet taste after dissolving. Fiber group 1 will take one bag (12 g) daily, while fiber group 2 will take two bags (24 g) daily, with both groups dissolving the fiber in warm water each morning.

### Criteria for discontinuing or modifying allocated interventions {11b}

Participants can withdraw from the trial or modify allocated interventions at any time for any reason. The investigator may discontinue patients from the trial to protect their safety or if they are unable or unwilling to continue with the trial (such as pregnancy occurs during trial participation). If adverse event is reported, doctors will also assess the patient to determine whether they should be withdrawn from the study or modify allocated interventions. The reason for each patient’s withdrawal, especially the occurrence of any adverse events, will be explained in detail.

### Strategies to improve adherence {11c}

Participants must return all the product containers every month to assess compliance and actual soluble dietary fiber intake. Adherence to the assigned treatments will be assessed through the use of a daily calendar during each phone call and by counting the returned packs at the end of the trial. Compliance rate will be calculated using the following formula: compliance rate = (packs taken/packs prescribed) × 100. A compliance rate below 80% will be classified as poor compliance.

### Relevant concomitant care permitted or prohibited during the trial {11d}

During the trial, participants will be prohibited from taking self-administered probiotics, dietary fiber supplements, antibiotics, or any other food/medication that may interfere with study outcomes.

### Participant timeline {13}

The protocol flow chart and timeline of the study are illustrated in Fig. 1 and, respectively.

### Sample size {14}

HbA1c monitors the exposure to circulating glycemia in the previous 3 months, whereas glycosylated serum protein (GSP) represents glycemia for 2–3 weeks [17–19]. Because this trial will last for 4 to 8 weeks, we chose the change in GSP as the primary endpoint, which may be more reliable. Since no previous studies assessed soluble fiber supplements for GSP in GDM patients, we calculated the sample size for HbA1c. In an inulin supplementation trial by Miao et al., a difference in HbA1c reduction of 0.27% was observed between the control group and the10 g/day inulin supplemented group [10]. We referred to the results of this trial, showing that an *n* = 37 would provide 90% power at an alpha level of 0.05. Allowing for a 20% dropout, a total sample size of 135 (45 per group) allows for an adequate sample size for our trial.

### Recruitment procedures {15}

Pregnant women who are diagnosed with GDM by the OGTT will be asked to undergo nutritional assessment at the nutrition clinic. After assessment, women who are potentially eligible to participate in our trial will be given a study flyer. At the same time, our principal investigator (Ping Gu) will explain the detailed information of the trial and screen for eligibility. The scientific value of this study and the potential benefits it may bring to patients will also be fully explained to potential participants to enhance their motivation for enrollment.

### Informed consent procedures {26a}

All participating patients will sign a written informed consent form. The principal investigator (Ping Gu) will explain the study’s purpose to the patients and obtain their written informed consent before they participate.

### Consent or assent: ancillary studies {26b}

We did not plan to collect participants’ data and biological specimens for ancillary studies.

### Allocation: sequence generation {16a}

Eligible patients will be randomized into three groups: The nutrition education group (*n* = 45, accept nutrition education of GDM), fiber group 1 (*n* = 45, receive nutrition education of GDM plus 12 g/day mixed soluble fiber, which contains 2 g of XOS and 10 g of inulin), and fiber group 2 (*n* = 45, accept nutrition instruction of GDM plus 24 g/day mixed soluble fiber, which contains 4 g of XOS and 20 g of inulin). Simple randomization with a 1:1:1 allocation ratio will be performed using a computer-generated random number sequence.

### Allocation: concealment mechanism {16b}

The allocation sequence will be concealed from those assigning patients to the intervention groups using opaque sealed envelopes.

### Allocation: implementation {16c}

An independent coordinator, who have no contact with participants, will generate a random value for each participant, shuffle the order based on random number sequence, and then assign them sequentially to one of three groups using numbered, opaque, sealed envelopes. Team investigators will access individual assignments by opening envelopes only after written consent is obtained. This method ensures a completely random and fair distribution.

### Outcomes {12}

The primary outcome measure will be the Change in GSP after 8 weeks of treatment across the different groups. GSP is a sensitive indicator of blood glucose levels in the last 3 weeks, making it particularly useful for monitoring pregnant women with diabetes. According to Morris et al., GSP levels correlate strongly with short-term glycemic control (fasting glucose: *r* = 0.798; postprandial: *r* = 0.846) and are measured via affinity chromatography targeting glycated serum proteins. This method allows frequent assessment of diabetic control, addressing the need for rapid treatment adjustments during pregnancy [20]. Secondary outcomes include fasting plasma glucose, 2 h-PPG, FINS, HbA1c, TC, LDL-C, HDL-C, TG, Wexner constipation score (WCS), the Bristol Stool Form Scale (BSFS), pregnancy outcomes, and changes in gut microbiota. Fasting venous blood will be collected at baseline (week 0), week 4, and trial completion (week 8). Blood samples will be processed within 2 h of collection (30-min room-temperature clotting, centrifugation at 3000 × g for 15 min), with serum/plasma aliquots stored at −80 °C. The Clinical Laboratory Department of Qingdao Municipal Hospital will test all the above laboratory indicators.

### Demographics and anthropometric measurements

We will collect information on demographic and anthropometric measurements, including date of birth, age, home address, telephone number, gestational age, height, weight, and body mass index (BMI). Blood pressure and heart rate tests will be conducted via an electronic blood pressure monitor. After the participants void their bladders and remove their heavy clothes and shoes, trained investigators perform a height and weight measurements via a studio meter and an electronic weighing scale. Participants will report their weight before pregnancy. BMI is calculated with an equation that incorporates height and weight: weight (in kg)/height^2^ (in m^2^) [21].

### FFQ and physical activities

To assess food and nutritional intake before and during the intervention period, experienced dietitians will Help participants complete the FFQ on day 0 and weeks 4 and 8. This questionnaire is a reasonably reliable and valid tool for assessing the food and nutrient intakes of urban pregnant women in Central China and consists of 10 food groups: cereals, meats, fish, eggs, beans, vegetables, fruits, nuts, milk or milk products, and beverages [22]. This tool requires participants to recall their usual frequency of intake and portion size during the past 4 weeks. Dietitians use color food photography atlas with different food portion sizes and food models to provide accurate estimate. After collecting the primary data, the daily nutrient intakes will be calculated based on the China Food Composition Database [23]. Participants will be asked about types and frequency of sports they play. Participants will be also recorded the average amount of time spent doing housework every day. The average walking time every day when they are on the way to and from work, working, shopping, and hanging out will also be recorded.

## Biological specimen collection, analysis, and storage

### Constipation assessment

Constipation is a common sign in GDM patients, and the outcomes from this trial may be beneficial for alleviating this disorder. Two scales, the Wexner constipation score (WCS) and the Bristol Stool Form Scale (BSFS), are used to assess the severity of constipation and measure stool form [24, 25]. The WCS is a Likert scale that includes eight items: the frequency of bowel movements, difficulty, completeness, pain, time, assistance, failure, and history. Based on the grading of the scale, scores range from 0 (normal) to 30 (severe), and a score of 15 is the cut-off point [26]. The BSFS is a 7-point scale for classifying stool forms into seven categories: separate hard lumps, such as nuts (hard to pass); sausage-shaped but lumpy; similar to sausage but with cracks on its surface; soft blobs with clear-cut edges (passed easily); fluffy pieces with ragged edges, a mushy stool; and watery, no solid pieces (entirely liquid) [25]. These two scales are widely used worldwide in clinical and research settings by doctors, researchers, and patients. Investigators will complete questionnaires based on the latest defecation status of the participants during three visits (weeks 0, 4, and 8).

### 16S rRNA gene sequencing and bioinformatics of the gut microbiota

In accordance with our protocol, the participants will be instructed to collect and store a sample of the first stool passed at home during the two visits (weeks 0 and 8). Sterile plastic containers with fecal samples will be sent to our nutrition clinic within 1 h after fecal collection. The samples will be transferred to the laboratory on dry ice within 48 h of collection and stored at − 80 °C until DNA extraction. DNA extraction kits (OMEGA Stool DNA Kit) will be used to extract gut bacterial DNA following the manufacture’s protocol. The PCR primer will be designed against the conserved region to target the variable region of the 16S rRNA gene. Microbiome signatures will be generated using the Illumina MiSeq platform barcoded V3–V4 primers targeting a hypervariable region of the 16S rRNA gene. The 16S rRNA gene amplification will employ the V3–V4 region-specific primers: 341F:5′-CCTACGGGNGGCWGCAG-3′ and 805R: 5′-GACTACHVGGGTATCTAATCC-3′. The V3–V4 primers (341F/805R) will be selected for their high taxonomic coverage (90–95% of gut taxa) and compatibility with sequencing platform (300-bp paired-end reads). It has also been proven effective in detecting microbiome shifts among GDM patients [27]. On the Illumina MiSeq platform, 300 bp × 2 paired-end reads will be generated.

After sequencing, paired-end reads will be assigned to samples based on their unique barcode and truncated by removing the index and primier sequences. Paired-end reads will be merged using Fast Length Adjustment of Short reads program (FLASH) [28]. Quality filtering of the raw reads will be performed under specific filtering conditions to obtain high-quality clean tags according to fqtrim [29]. Chimeric sequences were filtered using Vsearch [30]. After dereplication using Divisive Amplicon Denoising Algorithm 2 (DADA2) [31], the feature table and feature sequence will be obtained. A taxonomic classification will be assigned to amplicon sequence variants using the latest SILVA database with taxonomic classification at >99% confidence [32]. The community composition of gut microbiota will be counted at seven levels: kingdom, phylum, class, order, family, genus, and species. To explore each sample’s bacterial diversity and richness, alpha diversity, including the richness(observed species), Shannon indices, Chao 1, and Simpson indices,will be calculated. Beta diversity analysis will be performed to evaluate differences in the microbial communities among samples. Based on KEGG database, PICRUSt2 software will be applied to predict the functional profiles of the microbial communities and their pathways according to 16S rRNA gene sequencing data [33, 34].

### Pregnancy outcomes

After discharge, investigators will assess maternal and neonatal outcomes. Maternal outcome data collection will include premature delivery, hydramnios, proteinuria, cesarean, gestational age at delivery, postpartum Hemorrhage, and maternal OGTT results after 6 weeks. Neonatal outcomes are mainly related to the length of the newborn, birth weight, neonatal respiratory distress syndrome, Apgar score, and hypoglycemia.

### Adverse events {22}

Adverse events to be monitored include the following: Persistent diarrhea (defined as ≥ 3 loose stools per day for > 2 consecutive days); severe abdominal pain or bloating unresponsive to dose modification; clinically significant constipation (Wexner score ≥ 15 persisting after dietary intervention); confirmed hypersensitivity reactions (including but not limited to rash, angioedema, or dyspnea) attributable to XOS/inulin supplementation; hyperglycemia meeting diagnostic criteria (fasting plasma glucose > 7.0 mmol/L or 2-h postprandial glucose > 11.1 mmol/L on two consecutive measurements); new requirement for insulin therapy initiation; development of obstetric complications (preeclampsia, placental abruption, or other pregnancy-related emergencies); and hospitalization-requiring hyperemesis gravidarum or severe ketosis. Any adverse events should be reported to the researchers immediately. Investigators will also ask participants if they have any discomfort. All adverse events will be recorded in the participant case report forms. Once an adverse event is reported, doctors will assess the patient to determine whether they should be withdrawn from the study. All serious adverse events will be followed until resolution.

### Provisions for posttrial care {30}

Posttrial care will continue for 6-week postpartum, during which maternal OGTT results and infant growth/feeding data will be collected via telephone follow-ups. Nutritional counseling will remain available, but XOS inulin supplementation will cease after the 8-week trial period.

### Data collection plan {18a}

**Fig. 2:**
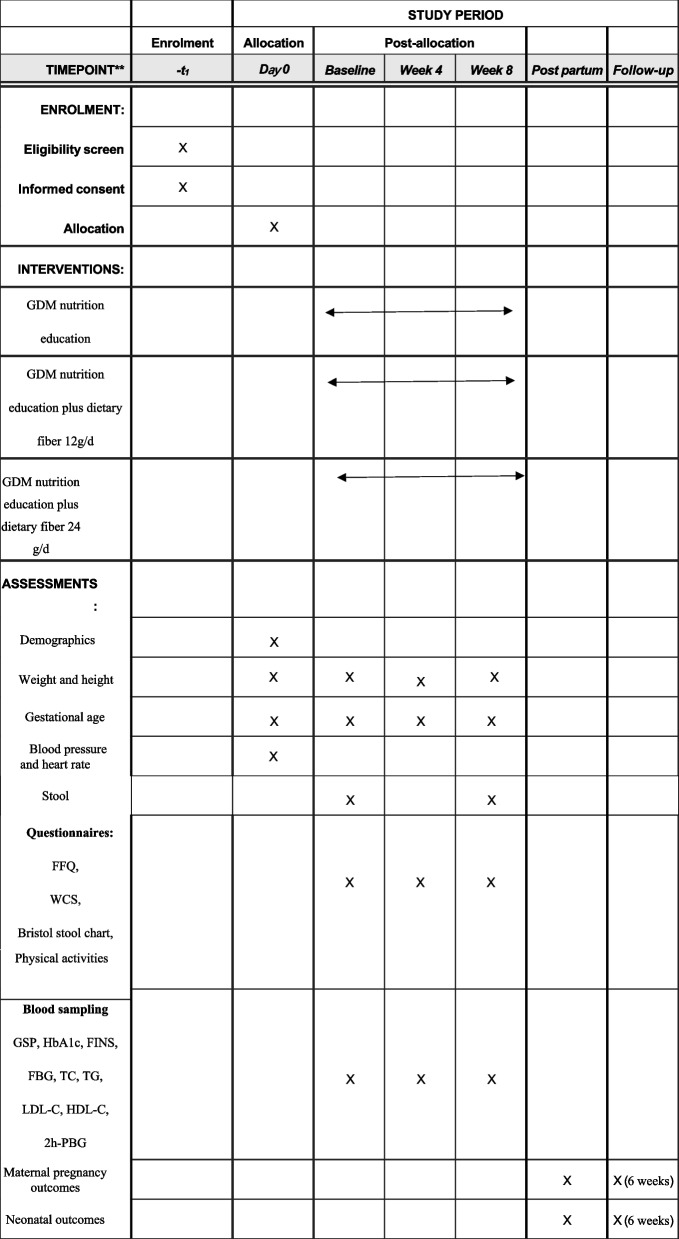
Standard Protocol Items: Recommendations for Interventional Trials (SPIRIT) schedule for enrollment, treatment, and assessments

Figure 2 shows the baseline and follow-up measurements and a summary of the data. The participant’s follow-up is scheduled for weeks 0, 4, and 8. In weeks 0 and 4, dietary fiber products were dispensed depending on the intervention groups. After obtaining informed consent, baseline assessments will be conducted, including demographics, gestational age, anthropometrics, blood pressure, and heart rate. During this visit, participants will have the first blood test (GSP, fasting glucose, fasting insulin (FINS), 2-h postprandial plasma glucose (2 h-PPG), HbA1c, total cholesterol (TC), LDL cholesterol (LDL-C), HDL cholesterol (HDL-C), and triglycerides (TG)). Trained dietitians will complete the study questionnaire, including a pregnant woman-specific food frequency questionnaire (FFQ), self-reported physical activities, the Wexner constipation score (WCS), and the Bristol stool chart. The same questionnaires and blood tests will be conducted at the second (week 4) and third (week 8) visits. Stool samples will be obtained from participants at every visit to analyze gut microflora colonization patterns. After delivery, information on maternal pregnancy outcomes and neonatal outcomes will be acquired through telephone calls and electronic medical system at the hospital. At 6-week postpartum, we will continue to collect maternal OGTT results and infant growth/feeding data through telephone follow-ups.

### Plans to promote participant retention and complete follow‑up {18b}

Participants will receive a call every week. We will inquire about and document the medication use during telephone visits. Dietitians will also communicate with participants via WeChat at all times to provide real-time assistance. They will also ask participants to document their 24-h dietary intake through photographs at least once a week, and food diaries will be analyzed using dietary analysis software. These approaches will improve participants’ adherence, promote participant retention, and ensure complete follow-up.

### Data management {19}

Research data will be coded as soon as possible using EpiData 3.1. To ensure quality, the data will be entered into EpiData with double-entry verification. Data will be available for researchers internal to the project at Qingdao Municipal Hospital, Department of Clinical Nutrition. They will have access to study protocol, statistical analysis plan, informed consent form, and database.

### Statistical analysis {20a}

Statistical analyses will be performed using the R 4.3.0 software. The Shapiro–Wilk test will be used to check for a normal distribution of the data. Continuous variables with a normal distribution will be presented as the mean (standard deviation [SD]); non-normal variables will be reported as the median (interquartile range [IQR]). Categorical data will be compared among the three groups using chi-square tests and partitions of the chi-square method. Linear models will be used to compare the mean values of the outcome variables among the three groups, adjusting for baseline values (e.g., age, BMI, nationality, gestational weeks, calorific intake). GSP Changes from baseline to week 8 will be analyzed using mixed-effects models with treatment group, time, and group × time interaction as fixed effects, adjusting for baseline GSP. *P*-value of less than 0.05 will be considered statistical significance. Post hoc pairwise comparisons were adjusted using the Bonferroni method to reduce the probability of type I error.

We will evaluate gut microbial diversity through complementary alpha- and beta-diversity analyses. For alpha diversity (within-sample microbial complexity), we will calculate three complementary metrics: Chao1 index (quantifying species richness), Shannon index (reflecting both richness and evenness), and Simpson index (measuring dominance of abundant species). Alpha-diversity differences between groups will be assessed using the Wilcoxon test. The cross-sectional difference in beta diversity (between-sample compositional differences) between the groups will be assessed by permutational analysis of variance (PERMANOVA) of Bray–Curtis dissimilarity and illustrated by PCoA models [35]. The significant bacterial taxa responsible for discrimination between the three groups will be identified via linear discriminant analysis (LEfSe) with linear discriminant analysis (LDA) [36]. Some multiple comparisons, such as differential abundance analysis of the microbiota, will be corrected using the Benjamini–Hochberg false discovery rate (FDR).

## Methods for additional analyses (e.g., subgroup analyses) {20b}

No subgroup analyses are planned.

### Methods in analysis to handle protocol non‑adherence and any statistical methods to handle missing data {20c}

The analysis will follow an ITT approach, which will include the data from the excluded patients in the results. Missing data will be addressed by multiple imputations. Sensitivity analyses will examine per-protocol completers.

### Composition of the coordinating center and trial steering committee {5d}

The coordinating center is the Department of Clinical Nutrition of Qingdao Municipal Hospital, which will assist in recruiting participants and collecting their questionnaires. The trial steering committee comprises four investigators who will supervise the trial and review its progress.

### Data monitoring: formal committee {21a}

The academic committee of Qingdao Municipal Hospital will perform impartial and continuous data monitoring. They will monitor data collection, trial conduct, patient safety, and efficacy, as well as to ensure the trial’s validity and integrity. It is independent from investigators and competing interests.

### Interim analyses {21b}

No interim analyses are planned for this trial.

### Auditing {23}

The ethics committee will monitor the quality, validity, and adherence to ethical standards throughout the study period, with at least two scheduled audits. In the event of ethical noncompliance or serious adverse events, corrective actions will be implemented, including potential study termination if warranted.

## Discussion

This paper describes the methodology of a clinical trial to assess the effectiveness of inulin and XOS in treating GDM patients. This trial will build on the relationship between inulin and XOS supplementation and blood glucose levels in GDM patients. Although current dietary strategies have result in positive outcomes for GDM patients, 10%–30% of patients still have to undergo medical treatment, such as insulin therapy [37, 38]. If a daily inulin and XOS intake favorably alters GDM glycemic control, fewer mothers will depend on insulin. As a strong predictor of the development of chronic noncommunicable diseases in the future, effective control of GDM will ease pressure on the health care system.

In this study, we will investigate potential correlations between microbiota changes and metabolic outcomes in GDM patients. Studies revealed that GDM patients exhibit significantly lower microbial gene counts and Shannon indices compared to healthy pregnant women [39–41]. A reduction in the SCFA-producing species *Bifidobacterium bifidum* and *Lactobacillus casei* has been reported in GDM patients [40]. XOS, an emerging prebiotic [42], was reported to result in a significant increase in the genus *Bifidobacterium* in in vitro fermentation and clinical trials [43, 44]. Although one study concluded that prediabetic individuals had significantly lower blood sugar levels after 8 weeks of XOS intake [45], its effects on GDM remain unclear. As a type of dietary fiber, inulin is fermented in the digestive tract, resulting in the formation of short-chain fatty acids (SCFAs) which lowers serum glucose and lipid levels [46, 47]. However, the results concerning the impact of inulin on glucose levels in individuals with type 2 diabetes are inconsistent [48, 49], and the evidence concerning the effects of daily inulin supplementation on pregnant women with GDM is insufficient. Our research will evaluate the therapeutic potential of combined XOS and inulin supplementation in GDM patients and explore potential associations between gut microbiota modulation and metabolic outcomes preliminarily through intestinal flora analysis.

One strength of this proposed study is that it may be the first clinical trial to examine the therapeutic effects of daily XOS inulin supplementation on pregnant women diagnosed with GDM. Two different dose groups and gut microbiota analyses are also considered strengths of this study. There are also several limitations of this study. First, women with gestational diabetes had an increased risk of developing type 2 diabetes compared with those who had a normoglycemic pregnancy [50], and the 8-week trial period cannot fully capture the long-term effects of XOS-inulin supplementation on glycemic control and pregnancy outcomes. Future studies with extended follow-up (e.g., postpartum or infant growth stages) are needed to evaluate sustained benefits, such as childhood obesity risk and type 2 diabetes mellitus after gestational diabetes. Second, as an open-label trial, the absence of a placebo group may introduce bias in participant-reported outcomes (e.g., constipation scores). Blinded designs could strengthen the validity of subjective measures. Third, the adherence was primarily monitored via self-report and product return counts, which may not fully reflect actual intake. Future trials should incorporate objective adherence markers (e.g., fecal XOS/inulin metabolites). Overall, all participants in this trial will be well-educated about GDM nutrition knowledge and behaviors, such as calculating meal energy values, understanding food nutrients, creating balanced meal plans, optimizing eating schedules, and breastfeeding/formula feeding best practices. We believe that our nutritional counselling will change their food habits and provide long-term health benefits [51].

## Supplementary Information {32}

Additional file 1. SPIRIT 2013 Checklist: Recommended items to address in a clinical trial protocol and related documents.

Additional file 2. Informed Consent Form.

## Abbreviations

GDM: Gestational diabetes mellitus
XOS: Xylose oligosaccharides
XOS inulin: Inulin and XOS
CMA: Chinese Medical Association
OGTT: Oral glucose tolerance test
GSP: Glycosylated serum protein
FINS: Fasting insulin
2 h-PPG: 2 h postprandial plasma glucose
TC: Total cholesterol
LDL-C: LDL cholesterol
HDL-C: HDL cholesterol
TG: Triglyceride
BMI: Body mass index
FFQ: Food frequency questionnaire
WCS: Wexner Constipation Score
FLASH: Fast Length Adjustment of short reads program
PERMANOVA: Permutational analysis of variance
DADA2: Dvisive Amplicon Denoising Algorithm 2
LefSe: Linear discriminant analysis
LDA: Linear discriminant analysis
SCFA: Short-chain fatty acids
