# Supplementary material for: Effects of soluble dietary fiber on glycolipid metabolism in gestational diabetes mellitus: study protocol for a randomized controlled clinical trial
Source: Trials. 2025 Sep 24;26:349. doi: 10.1186/s13063-025-09080-6 (PMC12462350; doi:10.1186/s13063-025-09080-6)
Supplement: Supplementary file 2 — Additional file 2. Informed Consent Form. [file 13063_2025_9080_MOESM2_ESM.docx]

**Informed Consent Form**

**Study Title**: Effects of Soluble Dietary Fiber on Glycolipid Metabolism in Patients with Gestational Diabetes Mellitus: A Randomized Controlled Clinical Trial
**Trial registration**: ChiCTR2200060117
**Version/Date**: V2.0/2022-03-31

**1. Study Purpose**

You are invited to participate in a research study evaluating the efficacy of XOS-inulin supplementation for gestational diabetes mellitus (GDM). This study is conducted by the Department of Clinical Nutrition at Qingdao Municipal Hospital and aims to explore new dietary approaches for improving glycemic control.

**2. What Will You Do?**

- **Group Assignment**: You will be randomly assigned to one of three groups (1:1:1 ratio):
  □ Nutrition education only
  □ Nutrition education + XOS-inulin (12 g/day)
  □ Nutrition education + XOS-inulin (24 g/day)
- **Follow-up**: The study lasts 8 weeks with 3 visits (weeks 0, 4, and 8), including:
  ✓ Blood tests (fasting glucose, HbA1c, etc.)
  ✓ Stool sample collection (gut microbiota analysis)
  ✓ Dietary records and questionnaires

**3. Potential Risks and Discomforts**

- **XOS-Inulin**: May cause bloating or diarrhea.
- **Blood Draw**: Minor pain or bruising.
- **Privacy Risks**: All data will be de-identified.

**4. Expected Benefits**

- Free professional nutrition guidance and metabolic monitoring.
- Potential improvement in your glycemic control.
- Contribution to scientific advances in GDM treatment.

**5. Prohibited Activities**

**During the trial, you must NOT**:
☒ Self-administer probiotics/dietary fiber supplements.
☒ Use glucose-lowering medications without approval from the study doctor.
☒ Modify the assigned intervention plan without approval.

**6. Voluntary Participation and Right to Withdraw**

- Participation is entirely voluntary. You may withdraw at any time without affecting your routine medical care.
- The study doctor may also discontinue your participation for safety reasons.

**7. Data and Privacy Protection**

- Your personal information will be encrypted and accessible only to the research team.
- No identifiable information will be disclosed in published results.

**8. Contact Information**

**Study Doctor**: Dr. Ping Gu, Department of Clinical Nutrition, Qingdao Municipal Hospital
**Phone**: +86-13954278272
**Ethics Committee**: Qingdao Municipal Hospital Ethics Committee (Approval No.: 2022Y006)

**Participant Declaration**:
"I have read and understood the above information, and my questions have been answered. I voluntarily agree to participate in this study."

Participant’s Signature & Date：

Investigator’s Signature & Date：
